# Renal sodium and magnesium reabsorption are not coupled in a mouse model of Gordon syndrome

**DOI:** 10.14814/phy2.13728

**Published:** 2018-07-20

**Authors:** Wouter H. van Megen, Paul R. Grimm, Paul A. Welling, Jenny van der Wijst

**Affiliations:** ^1^ Department of Physiology Maryland Kidney Discovery Center University of Maryland Medical School Baltimore Maryland; ^2^ Department of Physiology Radboud Institute for Molecular Life Sciences Radboud university medical center Nijmegen The Netherlands

**Keywords:** Gordon syndrome, kidney, magnesium, sodium

## Abstract

Active reabsorption of magnesium (Mg^2+^) in the distal convoluted tubule (DCT) of the kidney is crucial for maintaining Mg^2+^ homeostasis. Impaired activity of the Na^+^‐Cl^−^‐cotransporter (NCC) has been associated with hypermagnesiuria and hypomagnesemia, while increased activity of NCC, as observed in patients with Gordon syndrome, is not associated with alterations in Mg^2+^ balance. To further elucidate the possible interrelationship between NCC activity and renal Mg^2+^ handling, plasma Mg^2+^ levels and urinary excretion of sodium (Na^+^) and Mg^2+^ were measured in a mouse model of Gordon syndrome. In this model, DCT1‐specific expression of a constitutively active mutant form of the NCC‐phosphorylating kinase, SPAK (CA‐SPAK), increases NCC activity and hydrochlorothiazide (HCTZ)‐sensitive Na^+^ reabsorption. These mice were normomagnesemic and HCTZ administration comparably reduced plasma Mg^2+^ levels in CA‐SPAK mice and control littermates. As inferred by the initial response to HCTZ, CA‐SPAK mice exhibited greater NCC‐dependent Na^+^ reabsorption together with decreased Mg^2+^ reabsorption, compared to controls. Following prolonged HCTZ administration (4 days), CA‐SPAK mice exhibited higher urinary Mg^2+^ excretion, while urinary Na^+^ excretion decreased to levels observed in control animals. Surprisingly, CA‐SPAK mice had unaltered renal expression of *Trpm6*, encoding the Mg^2+^‐permeable channel TRPM6, or other magnesiotropic genes. In conclusion, CA‐SPAK mice exhibit normomagnesemia, despite increased NCC activity and Na^+^ reabsorption. Thus, Mg^2+^ reabsorption is not coupled to increased thiazide‐sensitive Na^+^ reabsorption, suggesting a similar process explains normomagnesemia in Gordon syndrome. Further research is required to unravel the molecular underpinnings of this phenomenon and the more pronounced Mg^2+^ excretion after prolonged HCTZ administration.

## Introduction

Serum magnesium (Mg^2+^) levels are kept within physiological range through the combined effects of intestinal uptake, storage in bone and urinary excretion by the kidney. Defects in either one of these components may result in hypomagnesemia (i.e., serum Mg^2+^ levels below 0.7 mmol/L) or hypermagnesemia (i.e., serum Mg^2+^ levels above 1.1 mmol/L) (de Baaij et al. 2015).

In the kidney, 70% of Mg^2+^ is freely filtered by the glomerulus and then selectively reabsorbed by different tubule segments along the nephron. The majority of Mg^2+^ reabsorption takes place in the proximal tubule and the thick ascending limb of Henle's loop, but the fine‐tuning occurs in the distal convoluted tubule (DCT) (de Baaij et al. 2015). The DCT reabsorbs Mg^2+^ from the pro‐urine through the apically located Mg^2+^ channel transient receptor potential melastatin type 6 (TRMP6), which is regulated at the level of transcription, plasma membrane abundance and activity (van der Wijst et al. 2014). In contrast to the well‐known apical site of entry, the basolateral mechanism of Mg^2+^ extrusion in the DCT remains largely unknown. Several candidate transporters have been postulated, including cyclin M2 (CNNM2) (Stuiver et al. 2011; Arjona et al. 2014) as well as solute carrier family 41 member 1 (SLC41A1) (Kolisek et al. 2012) and member 3 (SLC41A3) (de Baaij et al. 2016), but especially the role of SLC41A3 and CNNM2 as basolateral Mg^2+^ transporters remains controversial (Mastrototaro et al. 2016; Sponder et al. 2016).

The DCT is also an important site for sodium (Na^+^) reabsorption. Here, Na^+^ is reabsorbed through the apically located Na^+^‐Cl^−^‐cotransporter (NCC). The activity of this transporter, which facilitates electroneutral transport of Na^+^ and chloride (Cl^−^), is regulated through phosphorylation by the with‐no‐lysine kinase (WNK; i.e., WNK1 and WNK4)‐Ste20p‐related proline‐ and alanine‐rich kinase (SPAK) pathway (Richardson et al. 2008; Gamba 2012). Patients with loss‐of‐function mutations in the NCC‐encoding gene *SLC12A3* suffer from Gitelman syndrome (OMIM 263800) characterized by hypokalemia and hypomagnesemia (Gitelman et al. 1966; Simon et al. 1996). Pharmacological inhibition of NCC by long‐term treatment with thiazide diuretics, used to treat hypertension, is also associated with hypomagnesemia (Hollifield 1986; Davies and Fraser 1993; de Baaij et al. 2015). In addition, both SPAK^−/−^ and NCC^−/−^ mice develop hypomagnesemia (Schultheis et al. 1998; Yang et al. 2010; McCormick et al. 2011; Grimm et al. 2015; Verouti et al. 2015). These observations have led to the well‐accepted idea that Mg^2+^ reabsorption in the DCT is dependent on the activity of NCC and thiazide‐sensitive Na^+^ reabsorption.

However, increased NCC activity may not always be associated with increased Mg^2+^ reabsorption. In one human study, for example, NCC hyperactivity was not associated with hypermagnesemia (Mayan et al. 2002). A small number of patients with WNK4 mutations and Gordon syndrome (pseudohypoaldosteronism type II or familial hyperkalemic hypertension; OMIM 614491) who exhibited the classic signs of the disease (hypertension and hyperkalemia due to an increased activity of NCC (Gordon et al. 1970; Yang et al. 2003)), did not exhibit alterations in urinary or plasma Mg^2+^ (Mayan et al. 2002). Of note, this study only looked at eight unaffected and eight affected individuals within one family. Larger studies assessing plasma Mg^2+^ levels in patients with Gordon syndrome have currently not been performed.

To study the relationship between hyperactivation of NCC and renal Mg^2+^ handling, we investigated a mouse model of Gordon syndrome. This model was created by targeted DCT1‐specific knock‐in of constitutively active SPAK (CA‐SPAK) through phosphomimetic mutations at the key activation sites (T243E and S383D) (Grimm et al. 2017). These mice are characterized by NCC hyperactivity leading to hypertension and hyperkalemia, thus resembling the phenotype of patients with Gordon syndrome. The CA‐SPAK mouse model was used to determine urinary Mg^2+^ excretion and plasma Mg^2+^ concentrations, as well as gene expression of known magnesiotropic genes.

## Materials and Methods

### Animals and sample collection

The generation of the CA‐SPAK mice has been described previously (Grimm et al. 2017). In short, full length N‐terminal HA‐epitope SPAK cDNA bearing two mutations, T243E and S383D, which render the kinase constitutively active (Gagnon and Delpire 2010), was inserted after a floxed neomycin resistance gene cassette into the SPAK (STK39) gene. Successful insertion of construct creates a SPAK KO and allows CA‐SPAK to be expressed under control of the native SPAK promoter following recombination with Cre‐recombinase. To drive early DCT (DCT1)‐specific expression within the kidney, male mice homozygous for floxed CA‐SPAK were bred with female mice that express Cre recombinase under the control of the parvalbumin promoter (The Jackson Laboratory, B6.129P2‐Pvalb^tm1(Cre)Arbr/J^). DCT1‐specific CA‐SPAK (Parv‐Cre/CA‐SPAK) (*n *=* *14) and control mice, expressing the parvalbumin Cre‐driver alone (*n *=* *14), had ad libitum access to water and food. Mice were treated with an intraperitoneal (i.p.) injection of either hydrochlorothiazide (HCTZ; 25 mg/kg/ per day) or vehicle, once daily for 4 days. Subsequently, all mice were anesthetized by an i.p. injection consisting of 100 mg/kg ketamine and 10 mg/kg xylazine and their kidneys were collected. The cortex was subsequently isolated and stored in RNAlater (Qiagen GmbH, Hilden, Germany). Twenty‐four hour urine was collected at baseline and during each day of treatment. Blood samples were drawn from the carotid artery on day 4 after administration and immediately spun down to isolate the plasma. All animal experiments were performed in adherence to the NIH Guide for the Care and Use of Laboratory Animals and approved by the University of Maryland School of Medicine Institutional Animal Care and Use Committee.

### RNA isolation, cDNA synthesis, and qPCR

RNA was isolated from the cortex using an RNeasy Mini Kit (Qiagen GmbH, Hilden, Germany). RNA integrity was determined at the Core Facility of the University of Maryland School of Medicine using the RNA Integrity Number (RIN) method (Schroeder et al. 2006). Only samples with a RIN above 7.5 were deemed eligible. Subsequent cDNA synthesis was performed using SuperScript™ III Reverse Transcriptase (Thermo Fischer Scientific, Carlsbad, CA). Primer sequences of *Cnnm2*,* Fxyd2*,* Hnf1b*,* Slc41a1*,* Slc41a3*,* Trpm6,* and *Gapdh* are shown in Table 1 and were generated using MacVector version 7.2 (MacVector, Inc., Apex, NC). qPCR was performed using a Roche LightCycler 480 qPCR system (Roche Diagnostics GmbH, Mannheim, Germany) and LightCycler 480 SYBR Green I Master reagents (Roche Diagnostics GmbH, Mannheim, Germany). The analysis was performed using the 2^−ΔΔCt^ approach, using *Gapdh* transcript abundance for normalization and vehicle‐treated control littermates as the control group (Livak and Schmittgen 2001).

**Table 1:** Primer sequences used for gene expression analysis by qPCR

Gene	Forward primer	Reverse primer
*Trpm6*	5′‐AAAGCCATGCGAGTTATCAGC‐3′	5′‐CTTCACAATGAAAACCTGCCC‐3′
*Cnnm2*	5′‐AAGCACCCCAATGTCATCCAG‐3′	5′‐CATCACACCATAGTAGGAGAAAGCG‐3′
*Hnf1b*	5′‐GGCAAAAGAATCCCAGCAAGG‐3′	5′‐GAACCAGTTGTAGACACGGACCTC‐3′
*Fxyd2*	5′‐AGTGCCAAGGGGACAGAGAATC‐3′	5′‐CAGTTCCATCTTCATTGACCTGCC‐3′
*Slc41a1*	5′‐AAGTGCTGTTCCCCTTCCTACTG‐3′	5′‐CTGGGAACTCTACAGAAAAAGGGAG‐3′
*Slc41a3*	5′‐GCACGAGTCCTGCTCTTTCT‐3′	5′‐CACTTCTGCCAGGTACAGCA‐3′
*Gapdh*	5′‐TGATGGGTGTGAACCACGAG‐3′	5′‐GGCATGGACTGTGGTCATGA‐3′

### Urinary and plasma electrolyte measurements

Plasma and urinary samples were sent to IDEXX Preclinical Research Laboratories (Westbrook, ME) for analysis of plasma and urinary Mg^2+^ concentrations, as well as urinary concentrations of Na^+^. The 24‐h urinary Mg^2+^ and Na^+^ excretion was calculated using 24‐hour urine volume.

### Statistical analyses

All data are presented as mean ± SEM. Student's independent t‐test was used for statistical comparison; a *P* < 0.05 was considered statistically significant. All statistical analyses were performed using Graphpad Prism 5.0 (Graphpad Software). All experiments were performed in a blinded manner and unblinding occurred immediately before statistical analysis.

## Results

### CA‐SPAK mice exhibit normomagnesemia and no coupling of Mg^2+^ and Na^+^ reabsorption

**Figure 1:**
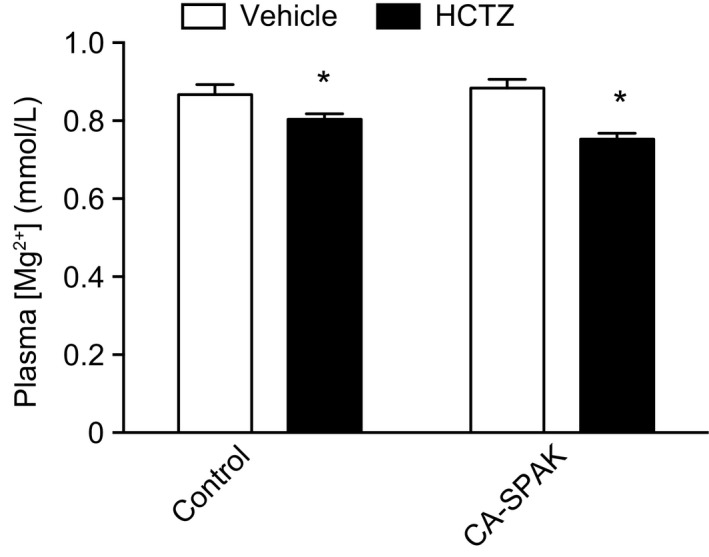
Plasma Mg^2+^ concentration for each group (*n* = 3). Blood samples were collected on the final day of either vehicle or HCTZ treatment for 4 days. Data are presented as mean ± SEM. *P < 0.05 versus vehicle‐treated group of the respective genotype.

**Figure 2:**
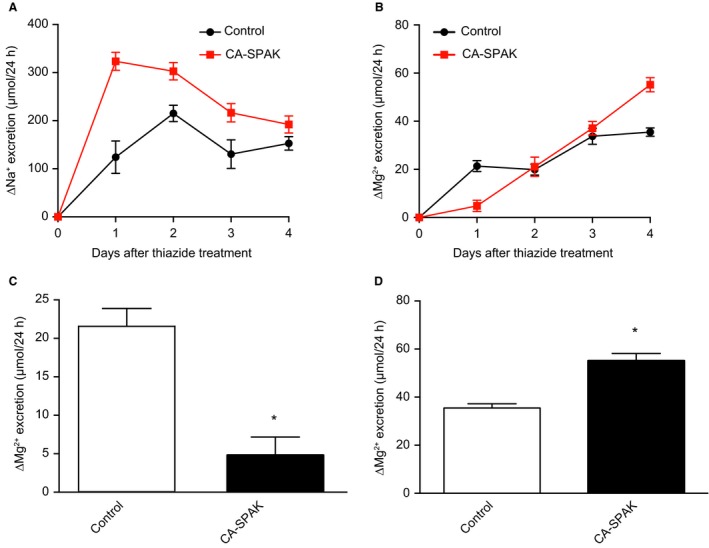
Change in urinary excretion of Na^+^ and Mg^2+^ in CA‐SPAK mice and control littermates after HCTZ treatment (*n* = 7 per group). Urine was collected on every day of HCTZ treatment. Data are presented as mean ± SEM and were calculated by subtracting Na^+^ and Mg^2+^ excretion values following HCTZ treatment with the excretion values of Na^+^ and Mg^2+^ at baseline, respectively. (A) HCTZ‐sensitive Na^+^ excretion and (B) HCTZ‐sensitive Mg^2+^ excretion is shown for control and CA‐SPAK mice. (C) Acute HCTZ‐sensitive Mg^2+^ excretion during peak HCTZ response (day 1). (D) Prolonged HCTZ‐sensitive Mg^2+^ excretion for both genotypes on day 4 of HCTZ treatment. * indicates *P* < 0.05 compared to HCTZ‐treated control mice.

To determine whether CA‐SPAK mice exhibit an altered steady‐state Mg^2+^ balance, plasma Mg^2+^ levels were determined. The CA‐SPAK mice had similar plasma Mg^2+^ levels as control mice (Fig. 1). Prolonged administration of HCTZ (4 days) induced hypomagnesemia in both groups, but there was no difference in response between the genotypes. To further investigate the observed normomagnesemia in CA‐SPAK mice, urinary Na^+^ and Mg^2+^ excretion was analyzed. No statistically significant differences were present in urinary Na^+^ or Mg^2+^ excretion at baseline, within or between genotypes (Table 2). As expected, and in concordance with a previous study (Grimm et al. 2017), CA‐SPAK mice showed higher HCTZ‐dependent Na^+^‐reabsorption compared to their control littermates, as indicated by a higher Na^+^ excretion following HCTZ treatment (Fig. 2A). Surprisingly, an opposite effect was observed on the Mg^2+^ excretion of CA‐SPAK mice (Fig. 2B). In fact, at the initial HCTZ response (day 1), Mg^2+^ excretion was significantly lower in CA‐SPAK mice (Fig. 2C), indicating that NCC‐ and/or SPAK‐dependent Mg^2+^ reabsorption is suppressed. Only until after longer treatment with HCTZ (4 days), did the CA‐SPAK mice display a significantly higher urinary Mg^2+^ excretion than control mice (Fig. 2B and D), consistent with indirect effects of HCTZ and/or NCC inhibition.

**Table 2:** Urinary Na^+^ and Mg^2+^ excretion at baseline and after treatment for each group (*n* = 7). Values are presented as mean ± SEM

Electrolyte	Mice group	Vehicle (*μ*mol/24 h)		HCTZ (*μ*mol/24 h)	
		Day 0	Day 4	Day 0	Day 4
Na^+^	Control	269.0 ± 15.3	275.7 ± 15.9	272.8 ± 18.4	425.5 ± 24.7
	CA‐SPAK	250.4 ± 6.6	277.7 ± 18.2	256.4 ± 11.2	448.4 ± 21.9
Mg^2+^	Control	27.2 ± 1.0	29.5 ± 1.7	29.5 ± 1.0	65.0 ± 1.4
	CA‐SPAK	27.2 ± 1.9	25.7 ± 0.7	26.9 ± 1.3	82.0 ± 2.1

### Expression of magnesiotropic genes

To explore the underlying mechanism of Mg^2+^ excretion in CA‐SPAK mice, the expression of genes known to be involved in Mg^2+^ reabsorption in the DCT was analyzed by qPCR. These include transcripts of *Trpm6*, transcription factor *Hnf1b* and *Fxyd2*,* Slc41a1*,* Slc41a3*, and *Cnnm2*.

**Figure 3:**
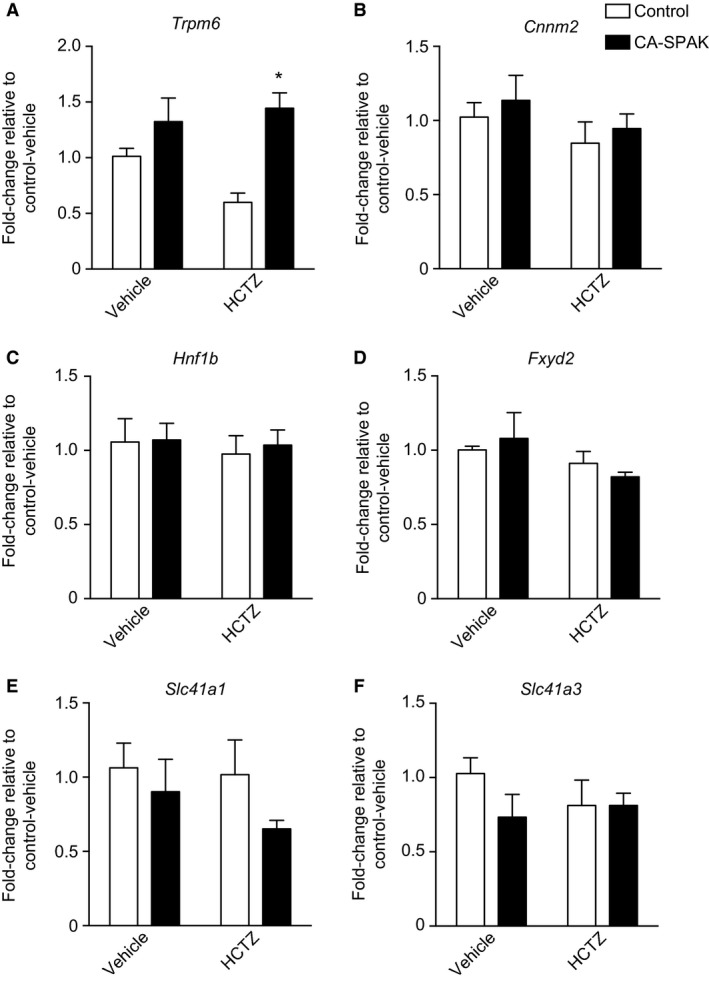
mRNA expression of known magnesiotropic genes expressed in the kidney as measured by qPCR. All measurements were performed after 4 days of thiazide or vehicle treatment. mRNA expression is shown relative to control littermates treated with vehicle and normalized using *Gapdh* expression for *Trpm6* (A), *Cnnm2* (B), *Hnf1b* (C), *Fxyd2* (D), *Slc41a1* (E), and *Slc41a3* (F). Data are presented as mean ± SEM. *indicates *P* < 0.05 compared to HCTZ‐treated control mice. *n* = 6 for vehicle‐treated control mice, *n* = 6 for vehicle‐treated CA‐SPAK mice, *n* = 7 for HCTZ‐treated control mice, and *n* = 5 for HCTZ‐treated CA‐SPAK mice.

The expression of these genes was compared between the vehicle‐treated control animals group and the vehicle‐treated CA‐SPAK group to determine whether CA‐SPAK mice have differential gene expression as a result of the constitutive SPAK signaling and NCC hyperactivity. No significant differences were present for renal expression of *Trpm6*,* Fxyd2*,* Slc41a1*,* Slc41a3*,* Hnf1b*, or *Cnnm2* (Fig. 3A–F). In addition, gene expression of the same genes was compared between HCTZ‐treated CA‐SPAK mice and control mice to investigate the higher Mg^2+^ excretion following chronic thiazide administration in CA‐SPAK mice. Expression of *Trpm6* was significantly higher in the 4 day HCTZ‐treated CA‐SPAK mice than in the HCTZ‐treated controls even though Mg^2+^ excretion was greater in the CA‐SPAK mice at this time point. In contrast, no significant differences were present for the other genes (Fig. 3A–F).

## Discussion

It is well established that decreased NCC activity is associated with urinary Mg^2+^ wasting and hypomagnesemia (e.g., Gitelman syndrome and chronic treatment with thiazide diuretics). This indicates that NCC‐dependent Na^+^ reabsorption is required for Mg^2+^ reabsorption in the DCT. Indeed, this notion is supported by the observation that SPAK^−/−^ mice, which lack the kinase responsible for phosphorylating NCC, also develop hypomagnesemia and renal Mg^2+^ wasting (Yang et al. 2010; McCormick et al. 2011; Grimm et al. 2015). However, patients with NCC hyperactivity (e.g., Gordon syndrome) are reported to be normomagnesemic and normomagnesiuric (Wilson et al. 2001; Mayan et al. 2002). Using a mouse model of Gordon syndrome, our study confirmed the normomagnesemic phenotype and revealed that this is due to the fact that thiazide‐sensitive Na^+^ reabsorption is not coupled to Mg^2+^ reabsorption. As estimated by the initial urinary response to HCTZ, we found NCC‐dependent Mg^2+^ reabsorption is actually suppressed in the CA‐SPAK mice, despite increased NCC activity. We speculate that enhanced Mg^2+^ reabsorption in other segments offsets the decrease in DCT‐specific Mg^2+^ reabsorption to maintain normal Mg^2+^ balance.

Since we did not observe any significant differences in the mRNA expression of magnesiotropic genes between control mice and CA‐SPAK mice under control conditions, we speculate that post‐transcriptional modifications are responsible for opposite responses on Na^+^ and Mg^2+^ reabsorption in CA‐SPAK mice. It is possible, for example, that constitutive SPAK signaling directly inhibits TRPM6, the apical Mg^2+^ channel responsible for Mg^2+^ reabsorption in the DCT. Technically demanding patch‐clamp experiments, in vivo or in vitro, on TRPM6 will be required to test this idea. In addition, the activities of other magnesiotropic proteins might also be contributory.

Because SPAK is the terminal kinase in the WNK signaling pathway that is altered in Gordon syndrome, our results should be broadly applicable to the disease. Nevertheless, it will be important to determine whether Mg^2+^ homeostasis is altered as a direct result of CA‐SPAK and whether a WNK4 mouse model of Gordon syndrome exhibits the same phenotype (Wilson et al. 2001). Interestingly, a WNK1 mouse model of Gordon syndrome, characterized by deletion of the first exon of *Wnk1*, reported normal plasma Mg^2+^ levels and urinary Mg^2+^ excretion (Vidal‐Petiot et al. 2013). A recent study by Terker et al. (2018) showed that WNK4 is involved in phosphorylation of the Na‐K‐Cl cotransporter 2 (NKCC2). Moreover, it was also shown that a WNK4‐Q562E mouse model of Gordon syndrome exhibited increased phosphorylated NKCC2 levels, which explained the normocalciuria in these mice. Since increased NKCC2 activity could lead to a more favorable electrical gradient for paracellular Mg^2+^ reabsorption (de Baaij et al. 2015), combined alterations in the Mg^2+^ handling in TAL and DCT may explain the normomagnesemia and normomagnesiuria in patients with Gordon syndrome.

It is also important to consider that the DCT1‐specific CA‐SPAK mice are engineered on a SPAK null background. Thus, when NCC is inhibited, these mice revert to a SPAK null phenotype. We (Grimm et al. 2015) and others (Yang et al. 2010; McCormick et al. 2011) have found that SPAK knockout mice exhibit a phenotype like Gitelman patients, who lack functional NCC (Gitelman et al. 1966; Simon et al. 1996), characterized by salt‐wasting, hypokalemia, and urinary Mg^2+^ wasting. The specific genetic background of CA‐SPAK mice could also have implications for renal Mg^2+^ handling in the thick ascending limb of Henle's loop given the data suggesting a role for SPAK in NKCC2 function (Moriguchi et al. 2005; Cheng et al. 2015). This may explain why CA‐SPAK mice excrete significantly more Mg^2+^ than control animals after prolonged HCTZ treatment. However, in contrast to NCC knockout mice and chronic HCTZ administration in wild‐type mice, which exhibit downregulation of *Trpm6* (Nijenhuis et al. 2005, we found *Trpm6* RNA levels are not affected in CA‐SPAK mice by prolonged HCTZ treatment. Thus, high levels of urinary Mg^2+^ excretion in the thiazide‐treated CA‐SPAK mice cannot be attributed to changes in *Trpm6* expression. We speculate that HCTZ‐induced atrophy of the DCT might be more pronounced in CA‐SPAK mice. This warrants additional experiments into alterations in tubular morphology following prolonged HCTZ treatment and their association with Mg^2+^ reabsorption.

## Conclusion

In summary, thiazide‐sensitive Na^+^ and Mg^2+^ reabsorption are not coupled in CA‐SPAK mice. These mice display an impaired NCC‐dependent Mg^2+^ reabsorption, despite constitutive hyperactivation of NCC. Like human subjects with Gordon syndrome, CA‐SPAK mice do not exhibit an altered Mg^2+^ balance. We speculate that enhanced Mg^2+^ reabsorption in other segments offsets the decrease in DCT to maintain normal Mg^2+^ balance. Further research is required to define the mechanisms.

## Conflict of Interest

The authors declare that there is no conflict of interest.
